# The development of multidisciplinary convalescence recommendations after childbirth: a modified Delphi study

**DOI:** 10.1016/j.xagr.2024.100411

**Published:** 2024-10-28

**Authors:** Zayël Z. Frijmersum, Eva Van der Meij, Esther V.A. Bouwsma, Corine J.M. Verhoeven, Johannes R. Anema, Judith A.F. Huirne, Petra C.A.M. Bakker

**Affiliations:** 1 Department of Obstetrics and Gynaecology, Research Institute, Amsterdam University Medical Center location Vrije Universiteit Amsterdam, Amsterdam, The Netherlands (Frijmersum and Huirne); 2 Amsterdam Reproduction and Development Research Institute, Amsterdam, The Netherlands (Frijmersum); 3 Department of Obstetrics and Gynaecology, Amsterdam University Medical Center location Academic Medical Center, Amsterdam, The Netherlands (Van der Meij, Huirne, and Bakker); 4 Department of Obstetrics and Gynecology, St. Antonius Ziekenhuis, Utrecht, The Netherlands (Bouwsma); 5 Midwifery Science, Amsterdam University Medical Center location Vrije Universiteit Amsterdam, Amsterdam, The Netherlands (Verhoeven); 6 and Division of Midwifery, School of Health Sciences, University of Nottingham, Nottingham, United Kingdom (Verhoeven); 7 Department of Public and Occupational Health, Amsterdam University Medical Center location Vrije Universiteit Amsterdam, Amsterdam, The Netherlands (Anema).

**Keywords:** Cesarean delivery, Childbirth, Convalescence Recommendations, Modified Delphi study, Multidisciplinary consensus, Outpatient recovery, Postpartum recovery, Vaginal delivery

## Abstract

**BACKGROUND:**

Evidence suggests that postpartum recovery takes longer than 6 weeks. However, evidence-based recommendations regarding postpartum recovery are lacking. Current research mainly focuses on shortening hospital stay after childbirth, neglecting outpatient recovery.

**OBJECTIVE:**

This study aimed to develop multidisciplinary recommendations on convalescence after vaginal and cesarean delivery using a modified Delphi method to improve recovery after childbirth.

**STUDY DESIGN:**

Multidisciplinary experts employed in different medical organizations involved in care and guidance of patients during postpartum recovery participated in the study. The panel included 16 experts (5 gynecologists, 2 senior residents, 4 midwives, 2 maternity nurses, 2 general practitioners, and 1 pelvic floor physical therapist) and representatives from medical organizations. Detailed recommendations on convalescence after uncomplicated vaginal delivery and uncomplicated cesarean delivery were developed. In addition, a list with 35 potential affecting factors that could delay recovery was presented to identify circumstances in which the convalescence recommendation should be adapted. Recommendations were based on a literature review and a modified Delphi procedure among 16 experts. Multidisciplinary consensus of at least 67% was achieved on convalescence recommendations for 27 relevant functional activities after childbirth.

**RESULTS:**

Multidisciplinary consensus on convalescence recommendations was reached for 26 of 27 functional activities for uncomplicated vaginal and cesarean delivery after 6 Delphi rounds and 2 group discussions. In total, 7 out of 32 affecting factors were deemed as independent factors that may delay recovery and therefore change the convalescence recommendations. The recommendations were deemed feasible by representatives from the same medical organizations as the panel.

**CONCLUSION:**

Multidisciplinary consensus on recommendations regarding convalescence after uncomplicated vaginal delivery and uncomplicated cesarean delivery was achieved.

**AJOG Global Reports at a Glance**

**Why was this study conducted?**

This study aimed to develop multidisciplinary recommendations on convalescence after vaginal and cesarean delivery using a modified Delphi method.

**Key findings**

Multidisciplinary consensus of at least 67% was achieved on convalescence recommendations for 27 relevant functional activities after uncomplicated vaginal delivery and cesarean delivery.

**What does this add to what is known?**

Multidisciplinary consensus on recommendations regarding convalescence following uncomplicated childbirth was achieved.

## Introduction

Evidence suggests that postpartum recovery takes longer than the expected period of 6 weeks.^1^^,^^2^ Studies have emphasized the importance of clarifying the recovery process; however, evaluation and recommendations regarding delayed postpartum recovery are still lacking.^3^^,^^4^ Given that a large group of theoretically healthy young women may experience delayed postpartum recovery and negative long- and short-term health effects, it is necessary to provide more information and guidance during this process.^1^^,^^5^^,^^6^

Most research has focused on reducing the length of hospitalization after cesarean delivery.^7^, ^8^, ^9^ Research on outpatient postpartum recovery is scarce, and evidence-based multidisciplinary convalescence recommendations regarding postpartum recovery are lacking.

Most healthcare professionals in obstetrics provide patients with experience-based recommendations, which often varies depending on the professional involved. Consequently, patients often receive inconsistent and contradicting advice leading to delayed recovery, which may result in poor well-being.^1^^,^^2^ This can have major socioeconomic consequences.^10^^,^^11^

Studies investigating the influence of postoperative advice on recovery suggest that uniform convalescence recommendations have a positive effect on early resumption of daily activities and work. However, there are no such recommendations for recovery from vaginal and cesarean delivery.^12^, ^13^, ^14^, ^15^, ^16^, ^17^, ^18^, ^19^ This underlines the need for accurate information about resumption of various activities following both cesarean and vaginal delivery.

To improve postpartum recovery and to provide a guiding tool for healthcare professionals and patients, development of multidisciplinary convalescence recommendations is essential. Therefore, the objective of this study is to develop multidisciplinary recommendations following vaginal and cesarean delivery using a modified Delphi method, with the aim to improve postpartum recovery.

## Methods

### Delphi study

We performed a Delphi study in which a multidisciplinary expert panel developed convalescence recommendations for the resumption of daily activities after childbirth. The Delphi method is proven to be effective for achieving consensus in different rounds when literature is insufficient or inconclusive.^18^, ^19^, ^20^

The aim of our Delphi study was to develop expert opinion in a systematic manner, supported by a systematic review of literature, on convalescence recommendations after vaginal and cesarean delivery.^21^ The experts in the panel were asked to anonymously complete questionnaires in repeated rounds and engage in group discussions to reflect on the results from previous rounds in a controlled manner. Successful completion of a Delphi round was achieved when consensus was reached according to previously defined consensus rules.

**Figure 1:**
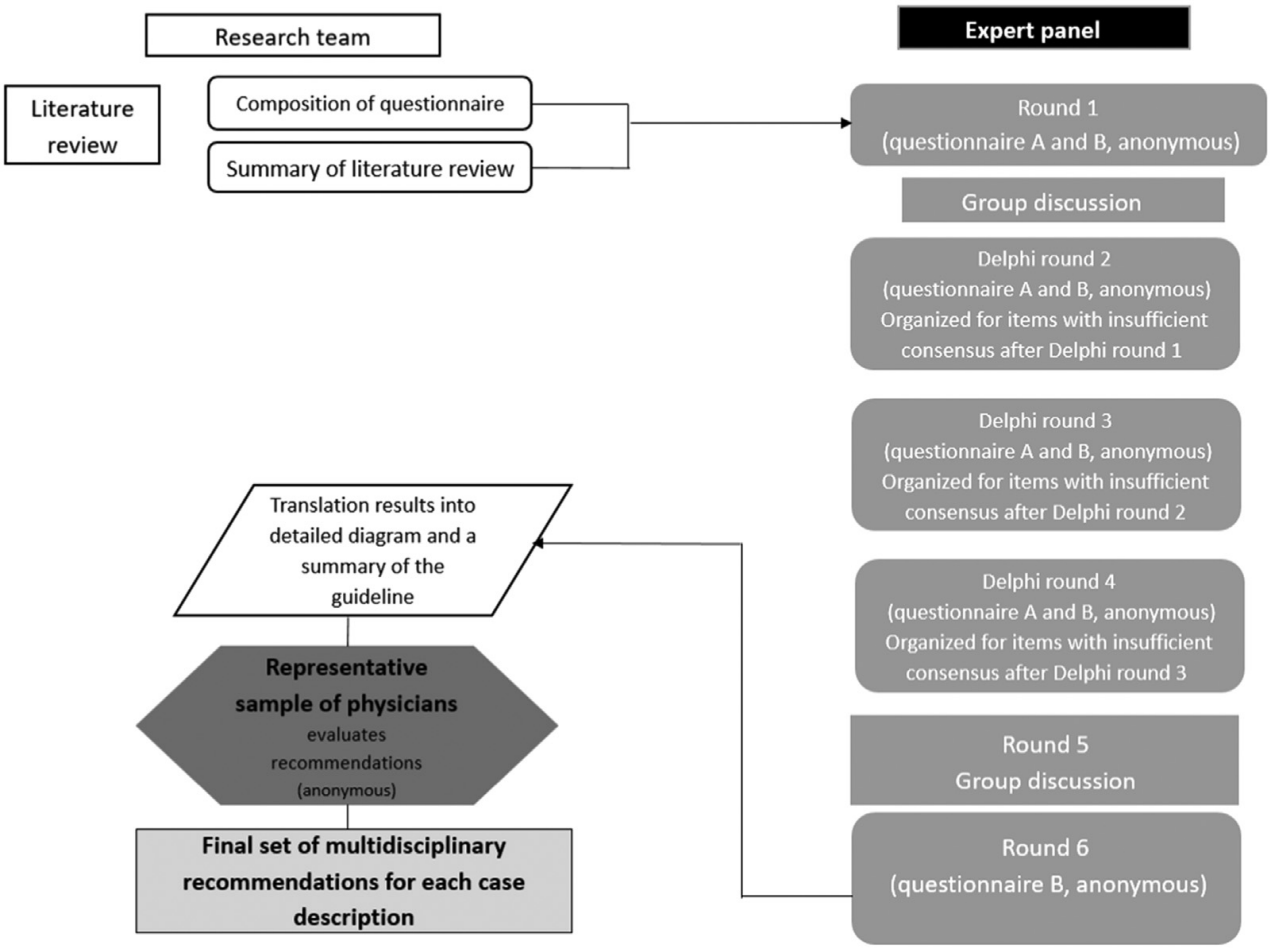
Study design

Data were collected between May 2021 and April 2022. The study design is presented in Figure 1.

Frijmersum. Convalescence recommendations after vaginal and cesarean delivery. Am J Obstet Gynecol Glob Rep 2024.

### Literature review

Similarly to previous Delphi studies on convalescence recommendations, we aimed to formulate evidence-based recommendations. Given that evidence is limited regarding this topic, we chose to select experts who could formulate convalescence recommendations based on literature and their professional experience. Therefore, an extensive literature search was performed in 5 databases (PubMed, Embase, Cochrane Library, CINAHL, and PsycInfo) to (1) evaluate current evidence regarding return to functional ability postpartum, including social activities and (2) identify potential factors affecting postpartum recovery. The following search terms were used, including free-text terms in the title or abstract, and MeSH (Medical Subject Headings) terms: “Childbirth,” “Delivery,” “Caesarean section,” “Recovery,” and “Time factors.” Studies were assessed for eligibility by 2 researchers (E.M., Z.Z.F.) according to a list of predefined inclusion criteria. These studies were provided to the Delphi panel members for reference during the Delphi rounds.

### Expert panel recruitment

To obtain a representative Delphi panel, we collaborated with professional medical organizations and asked which of their medical professionals wanted to partake in our expert panel. We aimed to include different types of healthcare professionals involved in the care and guidance of patients during their postpartum recovery, as they all have their own expertise. These healthcare professionals include gynecologists, gynecology residents, midwives, general practitioners, maternity nurses, and pelvic floor physical therapists. The gynecologists in our expert panel perform obstetrics as well as gynecology. In the Netherlands, gynecologists, gynecology residents, and midwives are responsible for the first 6 weeks of postpartum care. Maternity nurses are involved during the first 2 weeks after delivery, which can be extended until 6 weeks postpartum, and the involvement of pelvic floor physical therapists and general practitioners usually starts 6 weeks after delivery.

### Case definition

We formulated 2 obstetrical cases for which convalescence recommendations should be developed on the basis of the most prevalent modes of delivery: spontaneous vaginal delivery and elective cesarean delivery. Case descriptions for both cases were designed and were provided to the expert panel. The case description for spontaneous vaginal delivery consisted of a nulliparous patient who underwent an uncomplicated spontaneous vaginal delivery at approximately 40 weeks of gestation. For cesarean delivery, the case description consisted of a nulliparous patient with a fetus in breech presentation who underwent an uncomplicated elective cesarean delivery at approximately 40 weeks of gestation. Two different sets of recommendations were developed for each case description: medically justified advice (strong advice not to resume the activity sooner than recommended because it may be harmful) and realistic advice (at which moment will it be realistic to expect that a patient can perform a certain activity).

### Questionnaire development

We used the Functional Ability List (FAL)^22^ to develop the questionnaire, completed by the panel to develop the convalescence recommendations. The questionnaire is a standardized list and distinguishes 59 different physical and psychosocial activities, used by occupational and insurance physicians to assess and advise patients on their functional abilities in daily life.

**Figure 2:**
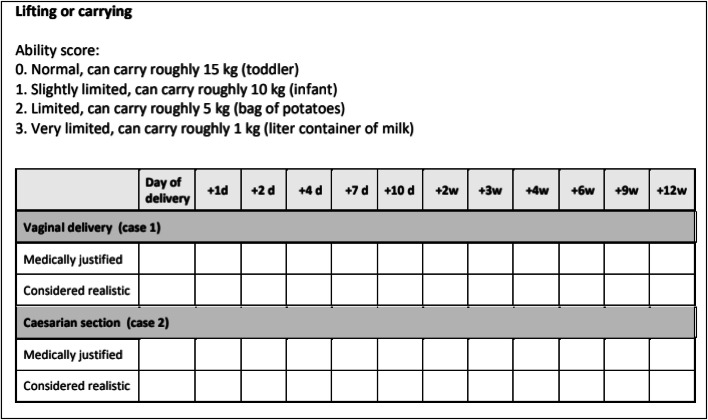
Example of Functional Ability List item “lifting or carrying”

We selected 27 activities from this list that were relevant for postpartum recovery recommendations according to previous studies.^18^^,^^19^ Activities could be dichotomous (2 possible answers: yes or no) or nondichotomous (>2 answers [eg, lifting: being able to carry 1, 5, 10, or 15 kg]). Figure 2 shows an example of an activity. This questionnaire was called questionnaire A. In addition, we created a list with 35 potential affecting factors (based on the literature review) that might delay recovery and therefore influence the convalescence recommendations for the 2 uncomplicated cases (questionnaire B).

Frijmersum. Convalescence recommendations after vaginal and cesarean delivery. Am J Obstet Gynecol Glob Rep 2024.

### Consensus rules

To determine which FAL items reached consensus and which did not (and thus had to be scored again in the next questionnaire round), we used a set of consensus rules.^18^^,^^19^ These rules were defined differently for dichotomous and nondichotomous activities. Consensus for dichotomous activities was reached when at least 75% of the expert panel agreed with the ability score on all time points. For activities with ≥3 categories of ability, consensus was reached when at least 66.7% of the expert panel agreed with the ability score given at the time points.

### Description of the structural consensus method

In the first Delphi round, panel members received questionnaire A and were requested to independently score the functional ability of each activity on the day of delivery until 12 weeks after delivery, divided in 11 to 14 time points. This resulted in visualizing the gradual resumption of the activity (Figure 2). In addition, each panel member assessed whether the factors listed in questionnaire B would affect recovery and thus change the convalescence recommendation for the activity, and if so, what the revised time frame might be. We provided background literature beforehand to assist in answering this questionnaire.

**Figure 3:**
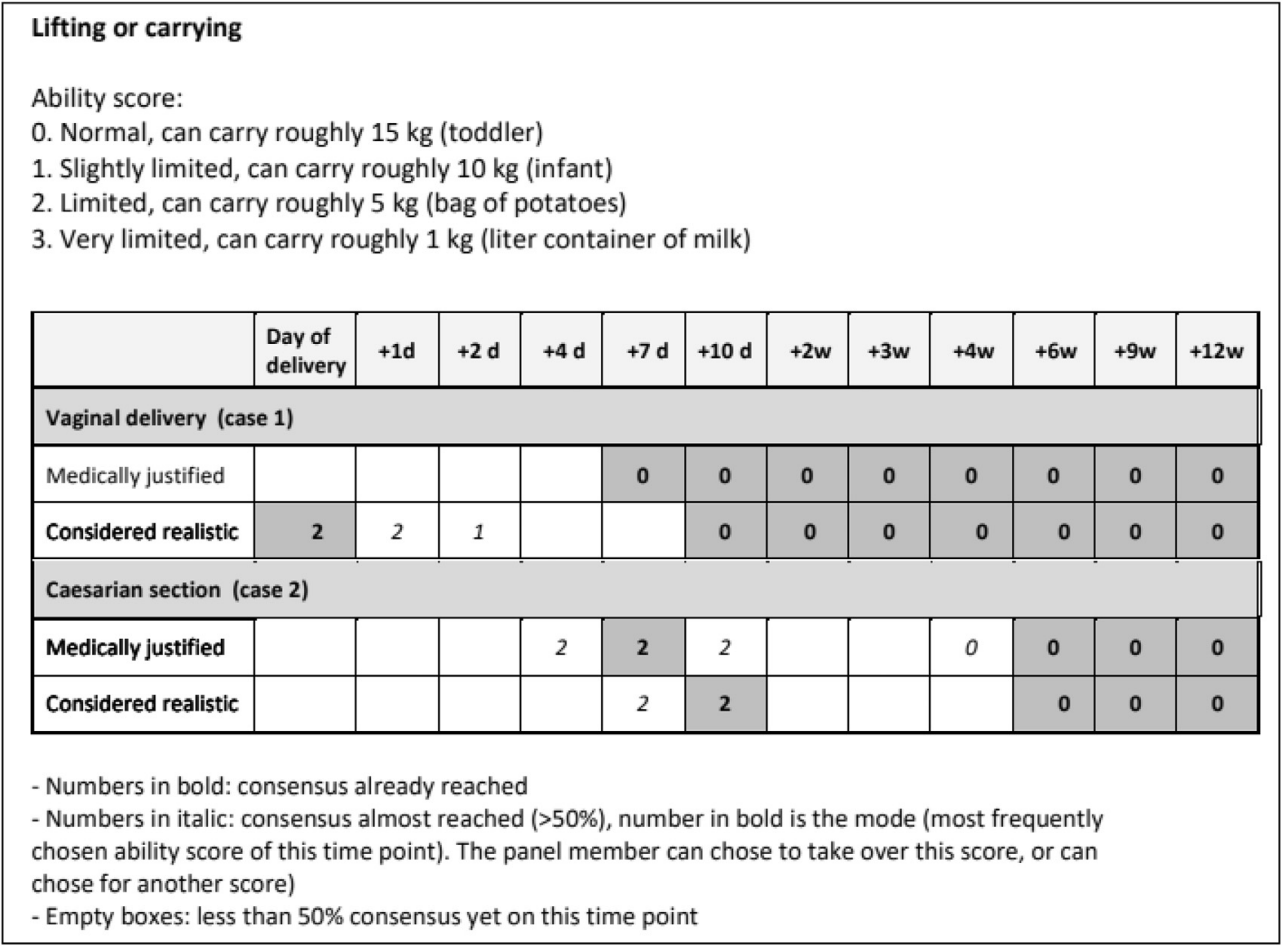
Example of the FAL item “lifting or carrying” in Delphi round 3 *FAL*, Functional Ability List.

After each round, the completed questionnaires were analyzed and consensus was evaluated by the research team. Subsequent questionnaires only consisted of FAL items and time points for which consensus had not been reached yet. From Delphi round 3 onward, if consensus for a FAL item at a specific time point was not reached but was already >50%, the mode for this time point was presented (Figure 3). The panel could decide if they agreed with the mode or if they had a different suggestion. This process continued until consensus was reached or until there was stagnation in reaching consensus. In case of stagnation, a group discussion was organized with an independent moderator. During this group discussion, the experts were able to discuss the FAL items. The mode values for each FAL item were also graphically presented during the discussion. Afterward, the experts were asked to rate the ability score for the specific FAL items again, taking into account the different opinions shared. Regarding questionnaire B, when the panel reached consensus on whether the additional factor would influence the ability score of a FAL item and therefore change the convalescence recommendation for both cases, the panel was asked in a subsequent Delphi round how this convalescence recommendation should change.

Frijmersum. Convalescence recommendations after vaginal and cesarean delivery. Am J Obstet Gynecol Glob Rep 2024.

The final result was a set of convalescence recommendations for the 2 cases on all FAL items included, and several additional convalescence recommendations for the specific affecting factors.

### Feasibility evaluation of the recommendations by a sample of physicians and patients

An overview of the convalescence recommendations created in the Delphi rounds was sent to 3 representatives of the same professional medical organizations as the expert panel, as well as 3 representatives of patient groups. They were asked to judge feasibility in daily practice.

## Results

### Literature review

After screening, 14 articles were selected as relevant for this study.^1^^,^^4^^,^^23^, ^24^, ^25^, ^26^, ^27^, ^28^, ^29^, ^30^, ^31^, ^32^, ^33^, ^34^ The articles were sent in full text to the panel members. These studies focused on functional recovery (n=3), recovery of physical activities (n=3), complicating factors that could delay recovery after childbirth (n=3), and sexual recovery (n=2). No articles on convalescence recommendations were identified.

### Expert panel

The panel consisted of 16 experts: 5 gynecologists (1.5–11 years of experience), 2 senior residents, 4 midwives (12–35 years of experience), 2 maternity nurses (5–13 years of experience), 2 general practitioners (5–6 years of experience), and 1 pelvic floor physical therapist (25 years of experience). All experts were of Dutch nationality. None had potential conflicts of interest.

### List of relevant convalescence recommendations

From the 56 activities of the FAL, a selection of 21 relevant items was made with the addition of 6 more activities (jumping, taking a bath, riding a bicycle, abdominal exercises, jogging, and sexual intercourse), resulting in a total of 27 activities for which convalescence recommendations were developed (Appendix A).

### Number of Delphi rounds and response rate

Six rounds, including 5 questionnaire rounds and 2 digital group discussions, were needed to meet the objectives of this study. The response rate for all rounds was 100%. All experts completed the entire study.

### Consensus course

#### First 4 Delphi questionnaire rounds and the 2 group discussions

After the first Delphi questionnaire round, consensus was not reached for any of the FAL items regarding both cases. The first group discussion took place after this round. The purpose of this group discussion was to give the panel the opportunity to ask questions regarding the method and provide additional information regarding the case descriptions. A second group discussion took place after 4 Delphi questionnaire rounds. After this group discussion, consensus was reached for 15 activities of questionnaire A and for all affecting factors of questionnaire B. Table 1 illustrates the flow of minimal consensus reached per individual time point of questionnaire A.

**Table 1:** Course of minimum consensus reached per individual time point for vaginal delivery

Consensus reached at every individual time point.

*FAL*, Functional Ability List.

#### Final Delphi questionnaire round

In the final Delphi questionnaire, FAL items that did not yet reach consensus were assessed. Afterward, consensus was reached for all items except one (“abdominal exercises”) at 6 weeks postpartum (Table 1). The study group concluded that consensus would not be reached for this activity at the time point.

Furthermore, the experts were asked to what extent, expressed in days, affecting factors would delay recovery and therefore influence the convalescence recommendations. The panel determined that third- or fourth-degree perineal ruptures, episiotomy, and postpartum hemorrhage (>2 L) would delay recovery for 3 activities and therefore influence the medically justified convalescence recommendations. Factors such as severe preeclampsia, emergency cesarean delivery due to fetal distress, and shoulder dystocia were found to delay recovery for the activities of concentrating and remembering, thus influencing realistic convalescence recommendations.

### Feasibility evaluation of recommendations by representatives of physicians and patients

For both cases, members of the same medical organizations as the expert panel and representatives of patient groups were asked to evaluate the convalescence recommendations within their respective groups. The recommendations were deemed feasible for implementation in daily practice. No revisions were requested.

### Final convalescence recommendations

For each case description (spontaneous vaginal and elective cesarean delivery), a definite set of convalescence recommendations was formulated, based on the results from the Delphi rounds.

Table 2 summarizes the recommendations for both cases, which can be used by healthcare professionals involved in recovery after childbirth. Additional recovery time for the agreed-upon factors affecting the convalescence recommendations are shown in Table 3.

**Table 2:** Summary of the convalescence recommendations

The resumption of activities is considered medically safe from the presented days/weeks after delivery. In case of a third- or fourth-degree vaginal tear or an episiotomy, the advice for sexual intercourse and riding a bicycle will be affected. In case of postpartum hemorrhage (>2 L), the advice for standing, walking, climbing staircases, and running will be affected.

^a^Allowed once vaginal bleeding has stopped.

**Table 3:** Affecting factors influencing convalescence recommendations by delaying recovery

Factor	Activity	Additional time^a^		
Grade 3/4 perineal rupture	Sexual intercourse	5 (2–6) wk		
	Riding a bicycle	17 (7–42) d		
		
Episiotomy	Sexual intercourse	5 (2–6) wk		
		For		
Postpartum hemorrhage >2 L	Sustained walking	1 h	15–30 min	5–15 min
	• Vaginal delivery	14 (1–21) d	7 (2–14) d	2 (1–7) d
	• Cesarean delivery	3 (2–5) wk	10 (7–18) d	4 (2–14) d
	Walking per day	Unrestricted	4 h per d	1 h per d
	• Vaginal delivery	3 (1–6) wk	10 (4–21) d	4 (2–14) d
	• Cesarean delivery	5 (2–6) wk	2 (3–5) wk	10 (5–14) d
	Climbing stairs	2 flights of stairs up or down	1 flight of stairs up and down	1 flight of stairs up and down
	• Vaginal delivery	4 (1–7) d	3 (1–7) d	1 (1–2) d
	• Cesarean delivery	11 (6–20) d	5 (4–10) d	3 (2–4) d

a Additional recovery time with regard to the convalescence recommendations shown in Table 2.

## Comment

### Principal findings

This study used a modified Delphi method to develop multidisciplinary, consensus-based recommendations on convalescence after spontaneous vaginal and elective cesarean delivery. After 5 Delphi questionnaire rounds and 2 group discussions, consensus was reached for 26 out of 27 activities (FAL list^22^). Consensus was not reached for 1 of the 14 time points for the activity “abdominal exercise.” Furthermore, 7 out of 32 affecting factors were evaluated as potentially delaying recovery and therefore influencing convalescence recommendations. Feasibility for daily practice was affirmed by representatives from the same medical professional organizations as the expert panel.

### Strengths and limitations

The primary strength of this study is the use of the modified Delphi method, incorporating a heterogeneous expert panel representing all healthcare professionals involved in maternity care, to provide expert opinions on postpartum recovery. This resulted in multidisciplinary consensus. The anonymous questionnaire rounds prevented bias. There were no dropouts, and all experts completed the Delphi procedure. Finally, the convalescence recommendations were deemed feasible by representatives from the same medical organizations as the expert panel and patient groups. Therefore, these recommendations are suitable for implementation in clinical practice.

A limitation of this study is the use of the FAL, originally developed for evaluation of functional ability by occupational and insurance physicians. Nevertheless, we used activities from this list to evaluate different gradations of strain after spontaneous vaginal and elective cesarean delivery. This method was used successfully in previous studies.^18^^,^^19^ Future research should consider confirming this instrument against validated instruments, such as the PROMIS (Patient-Reported Outcomes Measurement Information System) Physical Function.^35^ A second limitation is the size of our expert panel. To ensure the representativeness of our experts, we asked independent representatives of participating medical organizations to assess the recommendations for their applicability in daily practice. In addition, the study did not involve patient input during the questionnaire rounds because of the expected variations in patient experiences, as observed by Bouwsma et al.^36^ Nevertheless, we believe that the convalescence recommendations are scientifically grounded. The cases for which convalescence recommendations were formed were uncomplicated vaginal and elective cesarean deliveries, and further modifications to the convalescence recommendations are needed for pregnancies with high risk for complications. Finally, the developed recommendations might be incomplete regardless of the opportunities given to the expert panel and the representatives to provide suggestions for relevant activities. Future research will evaluate the developed convalescence recommendations in daily practice.

### Comparison with other studies

This Delphi study developed multidisciplinary recommendations for convalescence after spontaneous vaginal and elective cesarean delivery. No previous studies that developed or assessed postpartum convalescence recommendations were found. Existing guidelines indicate that physical activity during pregnancy is safe.^37^^,^^38^ Although some studies recommend resumption of physical activity postpartum, none provide specific recommendations regarding mode of delivery with corresponding time points.^23^^,^^39^^,^^40^ Uniform multidisciplinary convalescence recommendations regarding resumption of daily activities postpartum could improve maternal satisfaction and health.^40^

Previous successful modified Delphi studies from our study group for gynecologic, abdominal, and orthopedic surgeries underscore the effectiveness of the method.^18^, ^19^, ^20^ Collaboration among experts has led to consensus in relatively short periods, highlighting the effectiveness of the Delphi method.

In this study, consensus was achieved for most FAL items, except for “abdominal exercises” at 6 weeks postpartum, which is likely due to the lack of literature regarding postpartum resumption of physical activity. Our expert panel required 5 questionnaire rounds and 2 group discussions to reach completion of the study. The extensive process for reaching consensus reflects that postpartum recovery is poorly defined and insufficiently explored.

Our convalescence recommendations were similar to the recommendations developed for gynecologic surgeries (laparoscopic, vaginal, and abdominal) and abdominal surgeries (laparoscopic and open).^18^^,^^19^ For example, the expert panels agreed that it is medically safe to resume light activities after 2 days and to resume strenuous activities after 2 weeks following laparoscopic hernia repair, laparoscopic adnexal surgery, and spontaneous vaginal delivery. Likewise, resumption of light activities was deemed medically safe after 2 weeks and resumption of strenuous activities after 6 weeks following abdominal hysterectomy, open colectomy, and cesarean delivery. Previous studies implementing such recommendations in randomized controlled trials (RCTs) demonstrated improved patient outcomes without increased complications.^14^, ^15^, ^16^^,^^18^^,^^19^ The RCT by Vonk Noordegraaf et al^14^ found that patients who used an e-health intervention returned to work and normal activities sooner after hysterectomy and/or laparoscopic adnexal surgery compared with the control group. Specifically, the intervention group returned to work 9 days earlier than the control group.

In other RCTs, patients in the intervention group using a perioperative e-health program resumed normal activities 5 days earlier and returned to work 1 day earlier than the control group after intermediate-grade abdominal surgery. For major abdominal surgery, the intervention group resumed normal activities 13 days earlier and returned to work 2 days earlier than the control group.^15^^,^^17^ In addition, a positive effect was observed on social participation and physical function after use of the intervention program. Bouwsma et al^16^ support these findings in the context of different gynecologic surgeries.

These results suggest that implementing the recommendations of this study can yield similar outcomes. There is, however, a considerable difference between an elective surgical procedure and childbirth. Therefore, future studies are necessary to evaluate these recommendations.

### Interpretation of results and policy implications

The developed convalescence recommendations provide average recovery times for uncomplicated pregnancies and deliveries. Healthcare professionals can use them to guide patient recovery, with necessary adjustments for comorbidities or complications. With the introduction of enhanced protocols for recovery after cesarean delivery, standardized recommendations have become increasingly relevant.^7^, ^8^, ^9^ The use of such protocols is associated with reduced hospital stay, improved pain scores, shorter time to mobilization, and lower hospitalization costs. Therefore, our convalescence recommendations may increase self-management, prevent delayed outpatient recovery, and reduce short- and long-term negative health effects.^1^^,^^2^^,^^10^^,^^11^ However, maternal satisfaction and experiences require further evaluation.

### Future perspectives

To validate the developed multidisciplinary convalescence recommendations and assess prognostic factors, future research should conduct efficacy studies. In 2008, our study group developed a care program to optimize perioperative care by using e-health interventions to provide guidance from the preoperative phase until complete resumption of daily activities and work. Use of such e-health interventions resulted in faster return to work with a higher quality of life.^15^, ^16^, ^17^ These findings indicate the need for multidisciplinary convalescence recommendations.

We are currently designing a pilot study in which patients will be provided with convalescence recommendations through an e-health application after an elective cesarean delivery. The aim is to validate the convalescence recommendations and to monitor postpartum recovery. Future pilot studies will evaluate the convalescence recommendations after spontaneous vaginal deliveries.

## Conclusion

Consensus on convalescence recommendations following spontaneous vaginal and elective cesarean delivery was achieved by a multidisciplinary expert panel of healthcare professionals in obstetrics. These recommendations are the first step in improving postpartum recovery. Subsequent research will validate these convalescence recommendations and assess their efficacy in clinical practice.

## CRediT authorship contribution statement

**Zayël Z. Frijmersum:** Writing – review & editing, Writing – original draft, Project administration, Investigation, Formal analysis, Data curation. **Eva Van der Meij:** Writing – review & editing, Supervision, Project administration, Funding acquisition, Data curation, Conceptualization. **Esther V.A. Bouwsma:** Writing – review & editing, Methodology, Formal analysis, Conceptualization. **Corine J.M. Verhoeven:** Writing – review & editing. **Johannes R. Anema:** Writing – review & editing, Supervision. **Judith A.F. Huirne:** Writing – review & editing, Supervision. **Petra C.A.M. Bakker:** Writing – review & editing, Supervision.

## Appendix Supplementary materials

Appendix B: list of potential complicating affecting factors that may delay recovery Image, application 2
