# Supplementary material for: The development of multidisciplinary convalescence recommendations after childbirth: a modified Delphi study
Source: AJOG Glob Rep. 2024 Oct 28;4(4):100411. doi: 10.1016/j.xagr.2024.100411 (PMC11616063; doi:10.1016/j.xagr.2024.100411)
Supplement: Supplementary file 1 — Supporting information The following supplementary materials are available for this article: Appendix A: the functional ability list of the 27 items presented in Table 1 [file mmc1.docx]

**Appendix A – List of 27 activities for which convalescence recommendations are formed^1^**

| FAL-item | Max. number of existing gradations in which item is expressed |
| --- | --- |
| Bending over | 3 |
| Bending over frequently | 4 |
| Turning / twisting round | 2 |
| Pushing / pulling | 3 |
| Lifting or carrying^2^ | 4 |
| Handle light objects^3^ | 4 |
| Handle heavy objects^3^ | 2 |
| Walking – sustained^4^ | 4 |
| Walking – per day^4^ | 4 |
| Climbing flights of stairs | 4 |
| Kneeling or squatting | 2 |
| Sitting – prolonged | 4 |
| Sitting – per day^4^ | 4 |
| Standing – prolonged^4^ | 4 |
| Standing – per day^4^ | 4 |
| Actively kneeling or squatting | 2 |
| Actively turning / twisting around | 2 |
| Jumping | 2 |
| Taking a bath | 2 |
| Riding a bicycle | 2 |
| Abdominal exercises | 4 |
| Jogging / running (>6 km/h) | 4 |
| Driving a car^5^ | 2 |
| Sexual intercourse | 2 |
| Concentrating | 3 |
| Memorizing | 3 |
| Pace of action^6^ | 2 |

**^1^** FAL items for questionnaire A

^2^The activity of lifting or carrying a light or heavy object in general

^3^ The activity of frequently (10 times per minute or per hour) moving certain objects

^4^These activities should be considered in the context of active lifestyle or possible employment; when a person is required to perform any of these activities, it is important to know when resumption is advised.

^5^Driving after childbirth should be cleared with the insurance company

^6^The pace of action in relation to the demands set in life
