# Supplementary material for: The development of multidisciplinary convalescence recommendations after childbirth: a modified Delphi study
Source: AJOG Glob Rep. 2024 Oct 28;4(4):100411. doi: 10.1016/j.xagr.2024.100411 (PMC11616063; doi:10.1016/j.xagr.2024.100411)
Supplement: Supplementary file 2 — Appendix B: list of potential complicating affecting factors that may delay recovery [file mmc2.docx]

**Appendix B – List of potential complicating factors^1^**

| **Pregnancy related factors – These factors could potentially influence convalescence recommendations for both case descriptions** |
| --- |
| Pregnancy induced hypertension / mild preeclampsia |
| Severe preeclampsia / HELLP |
| Multiparity |
| Moderate to late preterm delivery (between 32 and 37 w) |
| Extremely to very preterm delivery (< 32 w) |
| Neonatal NICU admission |
| Neonatal admission transitional care unit |
| Indication for antibiotics during pregnancy |
| Twin pregnancy |
| **Personal factors - These factors could potentially influence convalescence recommendations for both case descriptions** |
| Episiotomy |
| Perineal rupture: 2^nd^ degree |
| Perineal rupture: 3^rd^ degree |
| Perineal rupture: 4^th^ degree |
| Neonatal birthweight > 4000 grams |
| Shoulder dystocia |
| Manual placental removal: incomplete placenta |
| Manual placental removal: no placenta |
| **Caesarean section – Factors potentially influencing convalescence recommendations for case description 2 (elective caesarean delivery)** |
| Caesarean section due to prolonged labour or fetal distress during labour |
| Caesarean section due to prolonged pushing (> 1 hour) |
| Caesarean section due to fetal distress during pushing (< 1 hour) |
| Previous caesarean section |
| **Factors potentially influencing convalescence recommendations for both case descriptions** |
| Postpartum haemorrhage 1000 ml – 2000 ml |
| Postpartum haemorrhage > 2000 ml |
| General anaesthesia |
| Breastfeeding |
| Maternal age > 40 years |
| Obesity (BMI 30 - 40) |
| Obesity (BMI > 40) |
| **Vaginal delivery – Factors potentially influencing convalescence recommendations for case description 1 (spontaneous vaginal delivery)** |
| Labour induction |
| Epidural analgesia |
| Morphine analgesia |
| Average dilation tempo < 1 cm per hour |
| Pushing phase > 1 hour |
| Ventouse delivery (1 traction) |
| Ventouse delivery (> 1 traction) |

^1^ Questionnaire B: list of affecting factors potentially delaying recovery and therefore influencing convalescence recommendations
